# Identification, Molecular Characterization, and Biology of a Novel Quadrivirus Infecting the Phytopathogenic Fungus *Leptosphaeria biglobosa*

**DOI:** 10.3390/v11010009

**Published:** 2018-12-25

**Authors:** Unnati A. Shah, Ioly Kotta-Loizou, Bruce D. L. Fitt, Robert H. A. Coutts

**Affiliations:** 1 Department of Biological and Environmental Sciences, University of Hertfordshire, Hatfield AL10 9AB, UK; unnatishah009@gmail.com (U.A.S.); b.fitt@herts.ac.uk (B.D.L.F.); r.coutts@herts.ac.uk (R.H.A.C.); 2 Department of Life Sciences, Imperial College London, London SW7 2AZ, UK

**Keywords:** fungal viruses, dsRNA mycoviruses, hypervirulence, *Leptosphaeria biglobosa* quadrivirus

## Abstract

Here we report the molecular characterisation of a novel dsRNA virus isolated from the filamentous, plant pathogenic fungus *Leptosphaeria biglobosa* and known to cause significant alterations to fungal pigmentation and growth and to result in hypervirulence, as illustrated by comparisons between virus-infected and -cured isogenic fungal strains. The virus forms isometric particles approximately 40–45 nm in diameter and has a quadripartite dsRNA genome structure with size ranges of 4.9 to 4 kbp, each possessing a single ORF. Sequence analysis of the putative proteins encoded by dsRNAs 1–4, termed P1–P4, respectively, revealed modest similarities to the amino acid sequences of equivalent proteins predicted from the nucleotide sequences of known and suspected members of the family *Quadriviridae* and for that reason the virus was nominated Leptosphaeria biglobosa quadrivirus-1 (LbQV-1). Sequence and phylogenetic analysis using the P3 sequence, which encodes an RdRP, revealed that LbQV-1 was most closely related to known and suspected quadriviruses and monopartite totiviruses rather than other quadripartite mycoviruses including chrysoviruses and alternaviruses. Of the remaining encoded proteins, LbQV-1 P2 and P4 are structural proteins but the function of P1 is unknown. We propose that LbQV-1 is a novel member of the family *Quadriviridae*.

## 1. Introduction

Fungal viruses or mycoviruses are ubiquitous and have been detected in all major groups of fungi including members of the divisions Ascomycota, Basidiomycota and Glomeromycota [1]. There are at least five established mycovirus families, and one established genus, whose members have multi-segmented dsRNA genomes: families *Reoviridae*, *Partitiviridae*, *Chrysoviridae*, *Quadriviridae*, and *Megabirnaviridae*, and the genus *Botybirnavirus* [1], which possess genome segment numbers of 11-12, 2, 4, 4, 2, and 2, respectively. In addition, there are two proposed families Alternaviridae and Polymycoviridae [2,3,4], which possess 4 and 4–8 genomic components, respectively. All of these viruses are encapsidated in rigid, spherical virus particles apart from polymycoviruses, which are not conventionally encapsidated [4,5]. Although many mycoviruses have no or few obvious effects on their host fungi, some induce phenotypic alterations including hypovirulence (attenuated virulence) or hypervirulence (enhanced virulence).

Phoma stem canker (blackleg) is an internationally important disease of oilseed rape (*Brassica napus*, canola, rapeseed), causing serious losses in Europe, Australia, and North America. For instance UK losses of ca. £100 million per season have been estimated using national disease survey data and a yield loss formula [6]. Phoma stem canker pathogen populations comprise two main species, *Leptosphaeria maculans*, associated with damaging stem base cankers, and *Leptosphaeria biglobosa*, often associated with less damaging upper stem lesions [6].

A collection of over 70 field isolates of *Leptosphaeria* spp. from *B. napus* were first classified as being either *L. maculans* or *L. biglobosa* and were then screened for the presence of dsRNA using a small scale isolation procedure. Several *L. biglobosa* isolates were found to contain dsRNA species whose electrophoretic pattern and sizes were reminiscent of those described previously for members of the *Quadriviridae* [7,8] and here we describe the complete molecular characterisation of a new quadrivirus isolated from a Chinese field strain of *L. biglobosa*. Moreover we carried out a complete analysis of the genome organisation and phylogeny of the virus, while its effects on the host phenotype and pathogenicity are described in detail elsewhere [9].

## 2. Materials and Methods 

### 2.1. Fungal Strains, Culture Conditions, and Dsrna Extraction

The Chinese *L. biglobosa* isolate W10 was grown on V8 agar plates and incubated for five days at 20 °C in darkness to produce confluent cultures. The isolate was confirmed as *L. biglobosa* following sub-culturing on potato dextrose agar (PDA) plates on the basis of morphological phenotype and by PCR amplification of the ribosomal RNA region incorporating the internal transcribed spacer (ITS) regions and the 5.8S rRNA gene [10]. The dsRNA elements known to be present in W10 [9] were purified by LiCl fractionation [11] and gel electrophoretic analysis of dsRNA was done according to standard protocols [4]. A virus-cured isogenic line was obtained using a combination of treatment with cycloheximide and hyphal tipping [4,9].

### 2.2. Virus Purification and Transmission Electron Microscopy (TEM)

Mycelia grown in PD broth with shaking at 25 °C were harvested five days after inoculation using sterile Miracloth (Merck Millipore, Danvers, MA, USA) and rapidly frozen in liquid N_2_ and kept at −80 °C until processing. Virus purification was performed as described before [5]. Purified virus was negatively stained with 1% uranyl acetate on carbon-coated 400-mesh copper grids and examined using a FEI Tecnai 20 transmission electron microscope. Isolation of dsRNA from purified virus was performed using phenol/chloroform treatment. DNase I (Promega, Madison, WI, USA) and S1 nuclease (Promega, Madison, WI, USA) treatments of purified dsRNAs were performed according to the manufacturer’s instructions.

### 2.3. cDNA Cloning and RNA Ligase-Mediated Rapid Amplification of cDNA Ends (RLM-RACE)

After electrophoretic separation on agarose gels dsRNAs were used, either collectively or individually, as templates for cDNA synthesis and PCR amplification of products using random priming, sequence-specific priming and RLM-RACE, which were subsequently cloned and sequenced [12,13]. At least three different clones were sequenced, covering the same part of each segment of the viral genome.

### 2.4. Sequence and Structure Bioinformatic Analysis

Sequence similarity searches of the GenBank, Swissprot and EMBL databases were conducted using the BLAST programs [14]. Searches for protein motifs were conducted using the Pfam database [15]. For phylogenetic analysis the RdRP protein sequences were aligned with MUSCLE as implemented by MEGA 6 [16], the alignment was improved manually and all positions with less than 30% site coverage were eliminated. Maximum likelihood phylogenetic trees were constructed in MEGA 6 using the LG + G + I substitution model. Structural models of the putative capsid proteins encoded by dsRNA 2 and dsRNA 4 were constructed using Phyre2 [17] and molecular graphics images were produced using the UCSF Chimera package from the Computer Graphics Laboratory, University of California, San Francisco (supported by NIH P41 RR-01081) [18].

### 2.5. SDS-Polyacrylamide Analysis of Purified Virus and Peptide Mass Fingerprinting (PMF)

Proteins obtained by the virus purification procedure from virus-infected strain W10 were analysed by gradient 4–15% SDS-PAGE on Mini-PROTEAN precast gels (BIORAD, Inc., Hercules, CA, USA) stained with the highly sensitive fluorescent SYPRO^®^ Ruby protein gel stain (Thermo-Fisher Scientific, Waltham, MA, USA) and visualised using the Fujifilm FLA-5000 Fluorescent Image Analyser. The PageRuler™ Prestained Protein Ladder (Thermo-Fisher Scientific, Waltham, MA, USA) was digitalised using EPSON Scan. Proteins were digested with trypsin and subjected to PMF broadly as described previously [7].

## 3. Results

### 3.1. Isolation of a dsRNA Mycovirus and Biological Comparison Of Virus-Infected And Virus-Free Isogenic Strains

**Figure 1:**
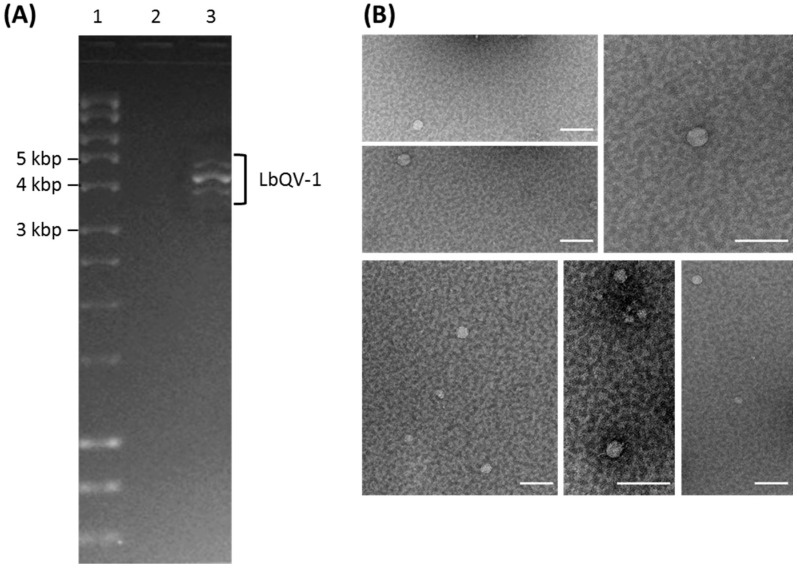
LbQV-1 particle morphology and dsRNA genome. (**A**) Agarose gel electrophoretic fractionation of the LbQV-1 genomic dsRNA segments. RNA was isolated from purified LbQV-1 preparations and the dsRNA profile of the virus-infected Chinese isolate W10 is shown in lane 3 while a dsRNA preparation from the same *L. biglobosa* isolate freed from virus infection and nominated strain W10-VF-1 is shown in lane 2. Lane 1 contains the DNA marker Hyperladder (Bioline), the sizes of which are shown to the left of the gel. (**B**) Electron micrograph of LbQV-1. Virus particles purified from *Leptosphaeria biglobosa* strain W10 were examined in the electron microscope after staining with uranyl acetate. Size bars indicate 100 nm.

Following extraction of dsRNA from the Chinese *L. biglobosa* isolate W10, it was discovered that it contained four dsRNA elements 4.9–4.0 kbp in size designated as dsRNA 1 to dsRNA 4, based on their gel mobility. Similar electrophoretic patterns have been reported for 10 more *L. biglobosa* isolates originating from China and the UK [9]. A representative agarose electrophoresis of the dsRNA elements isolated from *L. biglobosa* isolate W10 is shown in Figure 1A. As described previously, isolate W10 was successfully freed of infection with mycovirus dsRNAs using a combination of cycloheximide treatment and hyphal tipping to generate strain W10-VF-1 which was confirmed by electrophoretic isolation of dsRNA, Northern blotting and RT-PCR [9]. Comparison of the colony morphologies of the isogenic *L. biglobosa* W10 and W10-VF-1 lines revealed significantly different phenotypes associated with virus infection (Figure S1). A comparison of the mycelial growth rates of the two isogenic lines by examining radial expansion growth curves and biomass production emphatically demonstrated that infection of *L. biglobosa* with the mycovirus results in increased growth rate, as illustrated in Figure S1, and concomitant hypervirulence of the host fungus, phenomena which have been investigated in detail elsewhere [9].

### 3.2. Virus Particles, Genomic dsRNAs, and Their Sequences

Purified virus particles had an icosahedral structure and particle diameter was estimated to be ca. 40–45 nm (Figure 1B) and most particles were peripherally penetrated by the stain giving them a doughnut-like appearance. The chemical nature of purified preparations of dsRNA, either prepared directly from mycelia or isolated from virions, was confirmed by its insusceptibility to DNase 1 and S1 nuclease. The agarose gel electrophoretic patterns of the four dsRNA species with size estimates of 4.9, 4.4, 4.4, and 4.0 kbp were reminiscent of those seen for two members of the family *Quadriviridae*, *Rosellinia necatrix* quadrivirus strains W1118 and W1075, where the accumulation of dsRNA 1 and dsRNA 4 were also consistently less than those of dsRNA 2 and dsRNA 3 (Figure 1A). Based on these findings, the virus under investigation was nominated Leptosphaeria biglobosa quadrivirus-1 (LbQV-1).

A random cDNA library prepared from a mixture of LbQV-1 dsRNAs 1 to 4 was constructed. Approximately 50 clones with cDNA inserts of 500–1000 bp were selected, sequenced and assembled into contigs. The contigs covered almost the entire length of each dsRNA segment and gaps were filled by RT-PCR using oligonucleotides based on known sequences to generate amplicons, which were subsequently cloned and sequenced [4]. Terminal sequences of the four dsRNAs were determined by 3′-RLM-RACE and fragments for each end of both strands of the dsRNAs were cloned and sequenced. Because of heterogeneity at the extreme termini at least 12 clones were analysed, together with cloned internal amplicons and amplicons linking RACE clones with the assembled contigs.

**Figure 2:**
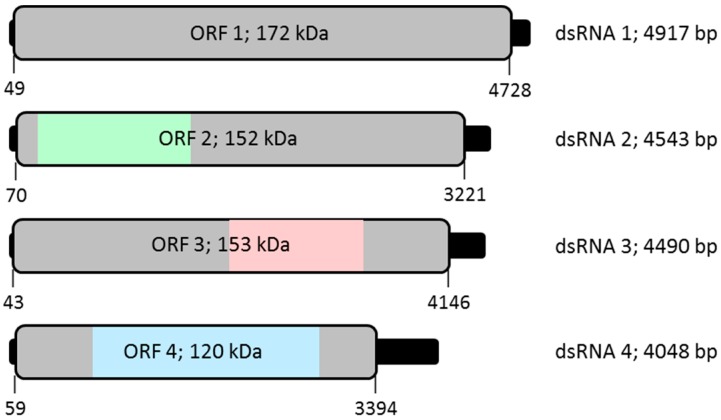
Schematic representation of the genomic organisation of LbQV-1. The LbQV-1 genome consists of four dsRNA segments, 4917, 4543, 4490, and 4048 bp in size, each containing one ORF shown as an open box flanked by 5′- and 3′-UTRs. A pink box on dsRNA 3 represents the RdRP domain. A blue and a green box on dsRNA 2 and dsRNA 4, respectively, illustrate the domains of the putative capsid proteins that were structurally modelled, as shown in Figure 4.

**Figure 3:**
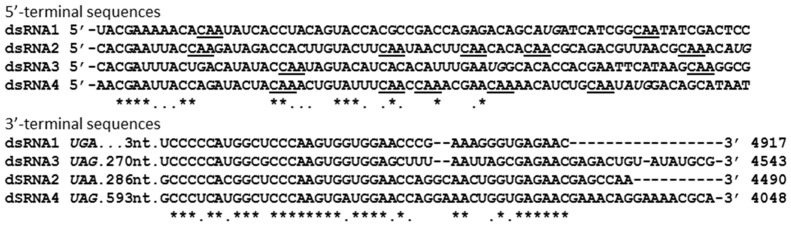
Terminal sequence domains of the LbQV-1 genome. Sequences of the 5′- (**A**) and 3′-termini (**B**). Identical sequences are denoted by asterisks and sequences present in ¾ dsRNAs are shown as dots. CAA repeats in the 5’terminal region are underlined and the possible initiation AUG codons and termination codons UGA, UAA and UAG are shown in italics.

The complete nucleotide sequences of the dsRNA 1-4 segments are 4917, 4543, 4490, and 4048 bp in size and each respectively contains an ORF on the plus-strand, potentially encoding proteins 1559 amino acids (aa; 172 kDa), 1383 aa (152 kDa), 1367 aa (153 kDa), and 1111 aa (120 kDa) in size, flanked by 5′- and 3′-untranslated regions (UTRs). Following the nomenclature adopted for other quadriviruses [7,8], these four proteins were nominated P1–P4. The 5′-UTRs of the four segments are respectively 48, 69, 42 and 58 bp long while the 3′-UTRs are 189, 322, 344 and 654 bp long (Figure 2; Table 1). Irrespective of which LbQV-1 dsRNA was used as template all 3′-RLM-RACE clones corresponding to the 5′-terminus of the plus-sense strand had sequences 5′-N/ACGA- (Figure 3) and are identical to the 5′-terminal sequences of the prototype *Rosellinia necatrix* quadrivirus strains W1118 and W1075 (RnQV1-W118 and W1075; [7,8]) and four large (L) dsRNAs isolated from plants infected with Amasya cherry disease (ACD-L), which probably represent the incomplete genomes of two closely-related quadriviruses of cherry or fungal origin [19]. However, the 3′-terminal sequences of the plus-sense strand of the LbQV-1 dsRNAs were highly variable and were extremely heterogeneous (Figure 3). Interestingly, however, there was significant homology between regions upstream of the 3′-termini of the LbQV-1 dsRNAs and these regions also contained characteristic sequence motifs including some small stem loop structures that were also identified in the 3′-UTRs of two isolates of RnQV1 and ACD-L dsRNAs 1–4. Heterogeneity at the extreme 3’termini is also a feature of the genomes of both RnQV1 and ACD-L dsRNAs 1–4.

### 3.3. Assignment of Structural Protein Genes

**Figure 4:**
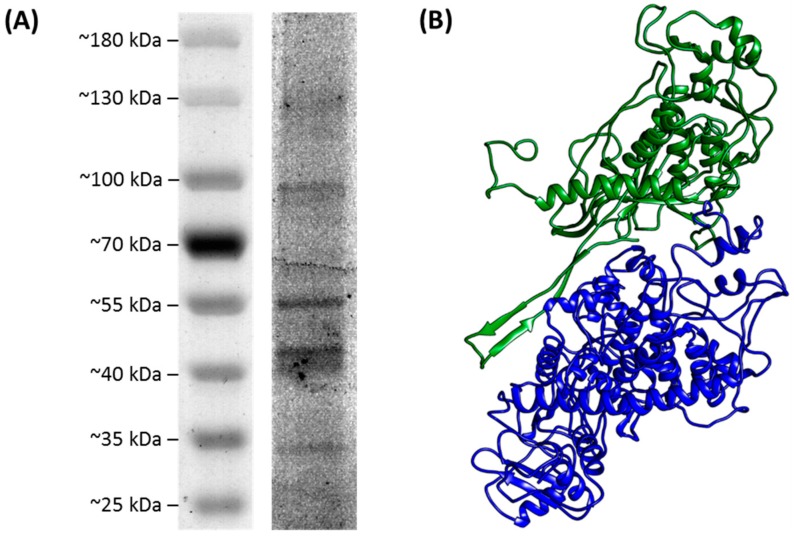
(**A**) SDS-PAGE pattern and modelling of LbQV-1 structural proteins. Proteins in a purified preparation of LbQV-1 were separated using a 4–25% gradient SDS-PAGE, stained with SYPRO^®^ Ruby and visualized by using the Fujifilm FLA-5000 fluorescent image analyser. The prestained protein ladder molecular weight sizes are shown to the left of the gel, following digitalization with EPSON Scan. (**B**) The partial structures and interaction between the two LbQV-1 structural proteins were predicted and visualized; the green and the blue protein domains are encoded by dsRNA 2 and dsRNA 4, respectively.

SDS-PAGE analysis of purified LbQV-1 showed the presence of two major proteins corresponding to ca. 130 kDa and 100 kDa as well as minor proteins of ca. 55 kDa, 40 kDa, and 35 kDa (Figure 4A). It is unclear whether all the observed proteins are viral since some of them may be derived from the host. Following PMF, four and six tryptic peptides were found to be derived from the N-terminal amino acid sequence encoded by dsRNAs 2 and 4, respectively (Table S1), verifying the presence of these proteins in the purified LbQV-1 preparation. Further PMF analysis of the proteins displayed on SDS-PAGE was not pursued but, by analogy with the full molecular characterization of the prototype quadrivirus RnQV1 [7,8], it can be assumed that the smaller proteins represent proteolytic degradation products of both proteins encoded by LbQV-1 dsRNA 2 and dsRNA 4 plus some host proteins. The known cryo-EM structure at 3.7 Å resolution of RnQV1 (PDBID 5ND1) was used as template for predicting the structure of the LbQV-1 putative capsid proteins and their interactions (Figure 4B).

### 3.4. Amino Acid Similarities and Phylogenetic Analysis

PSI-BLAST searches of the amino acid sequences of LbQV-1 P1–P4 generally showed modest similarities to the amino acid sequences of equivalent proteins predicted from the nucleotide sequences of known and suspected quadrivirus dsRNAs (Table 1). LbQV-1 P1 was distantly related to RnQV1-W1075 and RnQV1-W1118 (22% and 20% identity), ACD-L dsRNA 1 and ACD-L dsRNA 2 (26% and 27% identity) P1 equivalents and the sequence of one dsRNA component of a grapevine associated totivirus 1 (GaTV-1; 28% identity; [20]). Similarly a PSI-BLAST search with P2 showed only 23% and 22% amino acid sequence identity to equivalent proteins of RnQV1-W1118 and RnQV1-W1075. The results of PSI-BLAST searches with LbQV-1 P3 showed that it shared significant sequence similarity to known and suspected quadriviruses and was more closely related to the putative RdRPs encoded by ACD-L dsRNA 3 and -L dsRNA 4 (44% and 45% identity) and cherry chlorotic rusty spot (CCRS) L dsRNA 3 and -L dsRNA 4 (43% and 44% identity) as compared to RnQV1-W1118 and RnQV1-W1075 (35% and 36% identity). The PSI-BLAST search also showed that LbQV-1 P3 shares modest amino acid sequence identities of 20% to 30% to RdRPs from other mycoviruses within the families *Totiviridae*, *Chrysoviridae*, and *Megabirnaviridae* and an alignment of representative RdRP domains was generated (Figure S2; [21]). A Pfam search showed that LbQV-1 P3 belongs to the viral RdRP family (RdRp_4).

**Figure 5:**
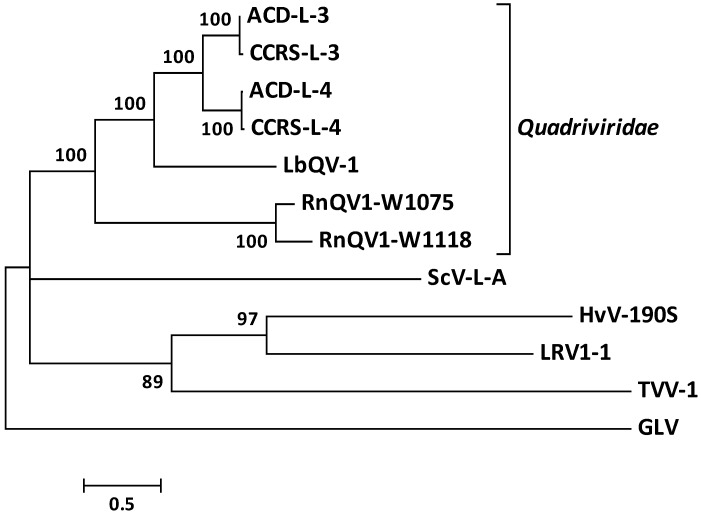
Phylogenetic analysis of Leptosphaeria biglobosa quadrivirus-1 (LbQV-1) RdRP and other known and suspected quadriviruses, together with selected members of the family *Totiviridae* based on the amino acid sequences of their RdRPs. The phylogenetic tree was constructed as described in the text. Only bootstrap percentages >50 are shown. Abbreviations and GenBank accession numbers for the sequences of the viral RdRPs used in the phylogenetic analysis are as follows: Amasya cherry disease-associated large dsRNA 3 (ACD-L-3; AM085134), Amasya cherry disease-associated large dsRNA 4 (ACD-L-4; AM085135), cherry chlorotic rusty spot-associated large dsRNA 3 (CCRS-L-3; AM181141), cherry chlorotic rusty spot-associated large dsRNA 4 (CCRS-L-4; CAJ57274), Rosellinia necatrix quadrivirus-1 strain W1118 (RnQV1-W1118; AB620063), Rosellinia necatrix quadrivirus-1 strain W1075 (RnQV1-W1075; AB744679), Saccharomyces cerevisiae virus L-A (ScV-L-A; J04692), Helminthosporium victoriae virus 190S (HvV-190S; U41345), Leishmania RNA virus 1-1 (LRV1-1; M92355), Trichomonas vaginalis virus-1 (TVV-1; U57898), and Giardia lamblia virus, GLV (L13218).

Sequences of representative RdRPs from fungal dsRNA viruses were used to construct maximum likelihood phylogenetic trees. As shown in Figure 5, the tree placed LbQV-1 P3 together with RdRPs encoded by ACD-L dsRNA 3, -L ds RNA 4, RnQV1-W1118, RnQV1-W1075, and GaTV-1 in a separate clade from other fungal dsRNAs. A PSI-BLAST search with LbQV-1 P4 showed only 24% amino acid sequence identity to equivalent proteins of both RnQV1-W1118 and RnQV1-W1075.

## 4. Discussion

The *Quadriviridae* is a family of non-enveloped spherical viruses with quadripartite dsRNA genomes of 3.5–5.0 kbp, comprising 16.8–17.1 kbp in total [22], which includes one genus with two strains of a single prototype species RnQV1 [7,8]. Here we have characterised a novel quadrivirus LbQV-1 from the filamentous fungus *L. biglobosa*, which shares some, but not all, biological and molecular features of the *Quadriviridae*. For instance, unlike RnQV1, LbQV-1 can be transmitted intracellularly via anastomosis and vertically through conidia, and it elicits alterations to the growth and pathogenicity of *L. biglobosa* resulting in the uncommon occurrence of hypervirulence (Figure S1; [9]). As with RnQV1, it is not known whether LbQV-1 has an extracellular phase. Transcription and replication are presumably carried out in a spherical particle by virion-associated RdRP.

Similar to RnQV1, LbQV-1 particles consist of rigid spherical virions ca. 40–45 nm in diameter (Figure 1B) which are encapsidated by two major structural proteins P2 and P4 encoded by dsRNA 2 and dsRNA 4, respectively (Figure 2). In both RnQV1 strains W1075 and W1118, the P2 and P4 proteins, which are more than 80% identical, form an asymmetric hetero-dimer subunit, and 60 of these build a T = 1 capsid [23]. We assume that LbQV-1 possesses a similar virion structure, having demonstrated moderate sequence identity between the equivalent proteins involved (Figure 2; Table 1). It is not known whether LbQV-1 P2 and P4 have acquired new functions through the insertion of complex domains at preferential insertion sites on the capsid outer surface as has been demonstrated for RnQV1 [23,24,25]. Both RnQV1 strains and LbQV-1 possess four linear dsRNA genome segments, termed dsRNA 1 to dsRNA 4 in a decreasing order of length from 5.0–3.5 kbp and each contains a single large ORF on the positive-sense strand of each dsRNA genomic segment (Figure 2). It is anticipated that each genomic dsRNA of LbQV-1 and both strains of RnQV1 are encapsidated separately [7,8]. LbQV-1, RnQV1-W1118, and RnQV1-W1075 genomic dsRNAs all had the same 5′-terminal motif sequence of 5′-N/ACGA- (Figure 3). The same motif is also present in the ACD-L dsRNAs, which probably represent the dsRNA 1 and 3 segments of two closely-related quadriviruses of cherry or fungal origin [19]. The lengths of all of the 5′-UTRs of fully sequenced quadrivirus dsRNAs are relatively small ranging in size from 35 to 106 nucleotides and those in both isolates of RnQV1 and LbCV-1 dsRNA 2 and 4 segments contain (CAA)_n_ repeats which are presumably translational enhancers, as reported for chrysoviruses [26]. Examination of the 3′-UTRs of the four dsRNA segments of two strains of RNQV1, LbQV-1 and ACD-L L reveal heterogeneity in the very terminal end sequences (Figure 3). However, as compared to RNQV1 and ACD-L the sequence heterogeneity found in all four LbQV-1 dsRNAs is extensive and regions of identity between them were only discovered some distance upstream of the very terminal end sequences (Figure 3). We believe that these observations for LbQV-1 and the discovery of an extraordinarily 645 nt long 3′-UTR for dsRNA 4 are not artefactual as we obtained the same results with a large number of representative clones in several different experiments. The occurrence of a large number of repeat sequences with the motif CA(A)_*n* = 2–8_ towards the 5′-terminus of the LbQV-1 dsRNA 4 3′-UTR is another novel feature for a quadrivirus the significance of which is unknown.

Sequence and phylogenetic analysis using the P3 sequence, which encoded the RdRP, revealed that LbQV-1 was most closely related to known and suspected quadriviruses and members of the genus *Totivirus* in the family *Totiviridae* [7,8] rather than other quadripartite mycoviruses, including chrysoviruses and alternaviruses (Figure 5; Figure S2; [7,8]). Of the remaining LbQV-1 encoded proteins, whereas LbQV-1 P2 and P4 are structural proteins the function of P1 is unknown. Based on the findings presented in this report, we propose that LbQV-1 is a novel member of the genus *Quadrivirus* in the family *Quadriviridae*.

## Figures and Tables

**Table 1:** Features of the LbQV-1 dsRNA segments 1–4 and comparisons with other known and suspected quadriviruses.

Segment	Virus	Nucleotide Sequence		UTR (nt)		ORF Properties		Accession Number
		Size (nt)	Identity (%)	5′-end	3′-end	Size (nt)	Size (aa)	
dsRNA1	RnQV1-W1075	4942	22	39	94	4809	1602	AB620061
	RnQV1-W1118	4971	20	39	126	4806	1601	AB744677
	ACD-L-1	5121	27	35	199	4867	1628	AM085136
	ACD-L-2	5047	26	35	199	4862	1620	AM085137
	GaTV-1	4218	28	91	-	-	850	GU108585
	LbQV-1	4917	100	48	189	4725	1559	LR028028
dsRNA2	RnQV1-W1075	4352	23	106	175	4071	1356	AB620062
	RnQV1-W1118	4307	22	41	195	4071	1357	AB744678
	LbQV-1	4543	100	69	322	4149	1383	LR028029
dsRNA3	RnQV1-W1075	4099	35	25	141	3933	1310	AB620063
	RnQV1-W1118	4093	35	23	140	3930	1309	AB744679
	ACD-L-3	4458	45	59	307	3992	1363	AM085134
	ACD-L-4	4303	44	73	345	3885	1294	AM085135
	LbQV-1	4490	100	42	344	4149	1367	LR028030
dsRNA4	RnQV1-W1075	3685	24	74	425	3186	1061	AB620064
	RnQV1-W1118	3468	24	45	246	3177	1058	AB744680
	LbQV-1	4048	100	58	654	3333	1111	LR028031

## Supplementary Materials

The following are available online at http://www.mdpi.com/1999-4915/11/1/9/s1, Figure S1: Colony morphology of virus-infected and virus-free *L. biglobosa* isolate W10 and W10-VF-1, respectively, on PDA plates following incubation at 20 °C for 26 days. Figure S2: Alignment of the region containing conserved motifs in the LbMV-1 dsRNA 3 RdRP with the RdRPs of some related dsRNA viruses. Table S1: Tryptic peptides derived from LbQV-1 P2 and P4 following PMF of the purified virus.
