# Supplementary material for: Identification, Molecular Characterization, and Biology of a Novel Quadrivirus Infecting the Phytopathogenic Fungus Leptosphaeria biglobosa
Source: Viruses. 2018 Dec 25;11(1):9. doi: 10.3390/v11010009 (PMC6356713; doi:10.3390/v11010009)
Supplement: Supplementary file 1 [file viruses-11-00009-s001.zip › SI/Figure_S1.docx]

**Figure S1.** Colony morphology of virus-infected and virus-free *L. biglobosa* isolate W10 and W10-VF-1 respectively on PDA plates following incubation at 20°C for 26 days. The photograph shows the appearance of the fungal colonies from the front (top) and the back (bottom) of the plates.
