# Supplementary material for: Identification, Molecular Characterization, and Biology of a Novel Quadrivirus Infecting the Phytopathogenic Fungus Leptosphaeria biglobosa
Source: Viruses. 2018 Dec 25;11(1):9. doi: 10.3390/v11010009 (PMC6356713; doi:10.3390/v11010009)
Supplement: Supplementary file 1 [file viruses-11-00009-s001.zip › SI/Figure_S2.docx]

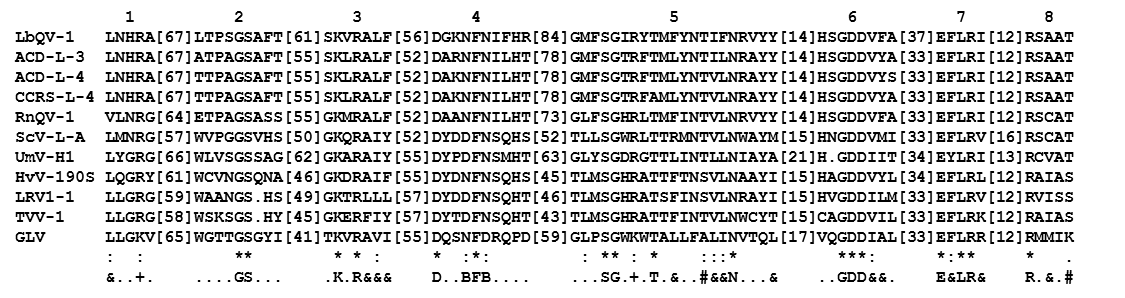


**Figure S2.** Alignment of the region containing conserved motifs in the LbMV-1 dsRNA 3 RdRP with the RdRPs of some related dsRNA viruses. The numbers of amino acid residues separating individual motifs are indicated for each sequence. The numbers at the top refer to the eight conserved motifs number [22]. In the RdRP consensus line, symbol ‘#’ signifies S or T, the symbol ‘&’ signifies hydrophobic residues (I, L, V, M, F, Y, W, C, A), the symbol ‘+’ signifies positively charged residues and ‘B’ signifies asparagine and aspartic acid. In the sequence alignment, asterisks signify identical amino acid residues, colons signify highly conserved residues, and single dots signify less conserved but related residues. Abbreviations and accession numbers of the viruses are shown in brackets: Leptosphaeria biglobosa quadrivirus, LbQV-1; Amasya cherry disease large dsRNA 3, ACD-L-3 (AM085134); Amasya cherry disease large dsRNA 4, ACD-L-4 (AM085135); cherry chlorotic rusty spot-associated large dsRNA 4 (CCRS-L-4; CAJ57274); Rosellinia necatrix quadrivirus 1, RnQV-1 (AB620063 and AB744679); Saccharomyces cerevisiae virus L-A, ScV-L1 (J04692); Ustilago maydis virus H1, UmV-H1 (U01059), Helminthosporium victoriae virus 190S, HvV-190S (U41345); Leishmania RNA virus 1-1, LRV1-1 (M92355); Trichomonas vaginalis virus-1, TVV-1 (U57898) and Giardia lamblia virus, GLV (L13218).
