# Supplementary material for: Identification, Molecular Characterization, and Biology of a Novel Quadrivirus Infecting the Phytopathogenic Fungus Leptosphaeria biglobosa
Source: Viruses. 2018 Dec 25;11(1):9. doi: 10.3390/v11010009 (PMC6356713; doi:10.3390/v11010009)
Supplement: Supplementary file 1 [file viruses-11-00009-s001.zip › SI/Table_S1.docx]

**Table S1.** Tryptic peptides derived from LbQV-1 P2 and P4 following PMF of the purified virus.

| **LbQV-1** | **peptide sequence** | **start (aa)** | **end (aa)** | **previous aa** | **next aa** |
| --- | --- | --- | --- | --- | --- |
| **P2** | ADNDLAEVTAMDASAR | 4 | 19 | K | V |
|  | VADTESK | 20 | 26 | R | S |
|  | SDLVSDSFDGNYGATR | 32 | 47 | R | V |
|  | VFQACNDVLK | 48 | 57 | R | H |
| **P4** | MDSIIDRL | 1 | 7 | - | L |
|  | LGSEGQIMTGVAK | 8 | 20 | R | H |
|  | HSINR | 21 | 25 | K | K |
|  | ACAEHDNAMELFSK | 31 | 44 | R | Q |
|  | NLATR | 48 | 52 | R | I |
|  | ITDNVVHG | 53 | 60 | R | M |
